# Supplementary material for: Mitochondrion-Targeted NIR Therapeutic Agent Suppresses Melanoma by Inducing Apoptosis and Cell Cycle Arrest via E2F/Cyclin/CDK Pathway
Source: Pharmaceuticals (Basel). 2022 Dec 19;15(12):1589. doi: 10.3390/ph15121589 (PMC9786161; doi:10.3390/ph15121589)
Supplement: Supplementary file 1 [file pharmaceuticals-15-01589-s001.zip › pharmaceuticals-2051462-supplementary.pdf]

Mitochondrion-targeted NIR therapeutic agent suppresses melanoma by inducing apoptosis and cell cycle arrest *via* E2F/Cyclin/CDK pathway

Changzhen Sun[1†], Jianv Wang[2†], Tong Xia[2], Qin Sun[1], Yijing He[3], Hailan Wang[1], Qizhou He[4*], Li Liu[2*]

[1] Drug Research Center of Integrated Traditional Chinese and Western Medicine, National Traditional Chinese Medicine Clinical Research Base, The Affiliated Traditional Chinese Medicine Hospital of Southwest Medical University, Luzhou, China.

[2] Department of Dermatology, The Affiliated Hospital of Southwest Medical University, Luzhou, China

[3] Department of Science and Technology, The affiliated hospital of Southwest Medical University, Luzhou, China

[4] Department of Radiology, The Affiliated Traditional Chinese Medicine Hospital of Southwest Medical University, Luzhou, China

† These authors contributed equally to this work.

* Corresponding author: liuli@swmu.edu.cn (L. Liu),
lyhqz0806@163.com (Q. He).

Tel: +86-193-3860-9127 (L. Liu); +86-159-0836-2735 (Q. He)

Table S1. Primer sequences for RT-qPCR assay.

	Primer Name	Sequence (5'-3')
108961	*E2f8-F*	GCTCTGAAGGAGGATTGACAGG
	E2f8-R	ATGCAGGGGGCTTTTCATCA
52679	*E2f7-F*	GATGCGTTCGTGAACTCCCTG
	E2f7-R	CCGACGACACGGTCTTCAAAGA
12544	*Cdc45-F*	CCGGCAACAAGGAACCAATC
	Cdc45-R	GGCGGATACCTAGAACTGGC
12428	*Ccna2-F*	GAGCTCCCAAGCTCTACTGC
	Ccna2-R	TTTTCATGGGCAGTCCTGGT
12534	*Cdk1-F*	AAGTGTGGCCAGAAGTCGAG
	Cdk1-R	TCGTCCAGGTTCTTGACGTG
20135	*Rrm2-F*	TGATGCCGGGCCTTACATTT
	Rrm2-R	CCTGCTCTATCCTAACGGCG
12433	*Ccnd1-F*	CAGCCCCAACAACTTCCTCT
	Ccnd1-R	CAGGGCCTTGACCGGG
12567	*Cdk4-F*	CTTAGCCGAGCGTAAGGCTG
	Cdk4-R	CCAGGCCGCTTAGAAACTGA
12447	*Ccne1-F*	GACACAGCTTCGGGTCTGAG
	Ccne1-R	CTGGAGCGGACTGAAAGGTC

12566	***Cdk2-F***	CGGCTCGACACTGAGACTG
	Cdk2-R	TTCTTGAGGTCCTGGTGCAG
12575	***P21-F***	CAGAATAAAAGGTGCCACAGGC
	P21-R	CGTCTCCGTGACGAAGTCAA
12576	***P27-F***	CAGACGTAAACAGCTCCGAATTA
	P27-R	GGCAGATGGTTTAAGAGTGCC

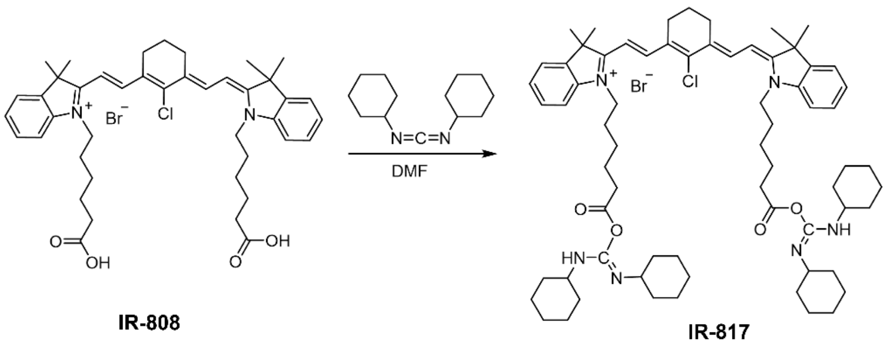

Figure S1. The synthetic route of IR-817.

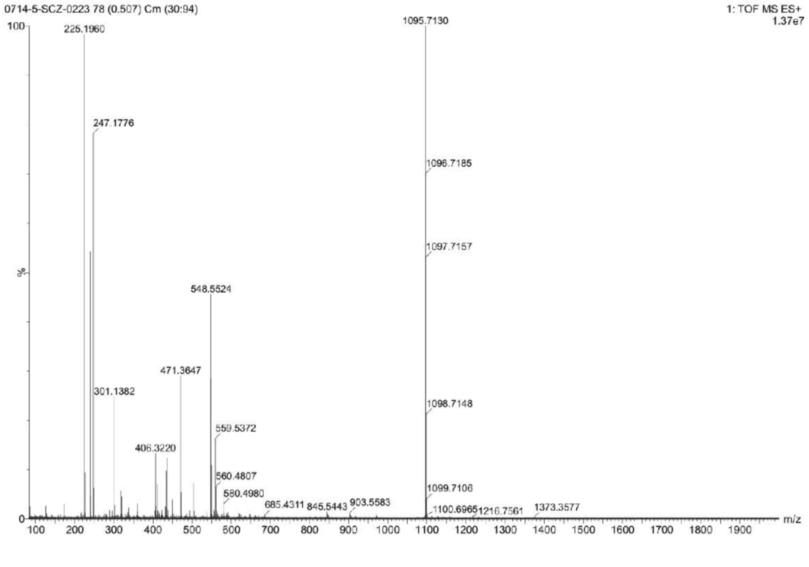

Figure S2. HRMS spectrum of IR-817.

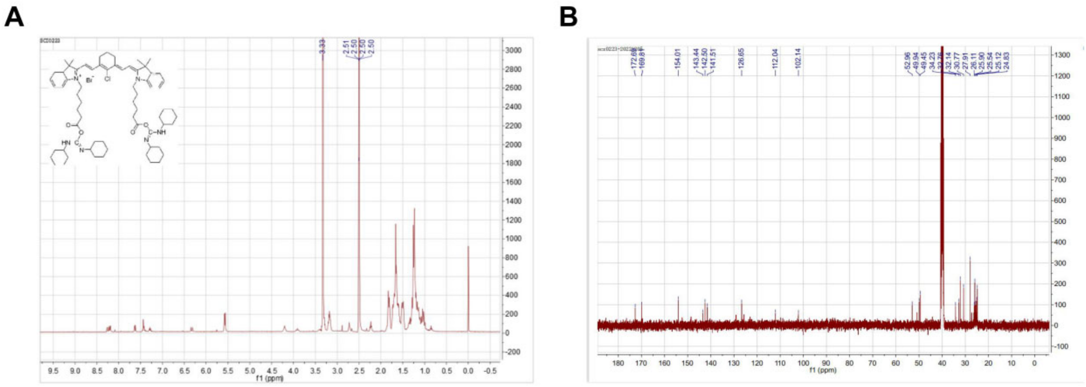

Figure S3. ^{1}H NMR and ^{13}C NMR spectrum of IR-817.

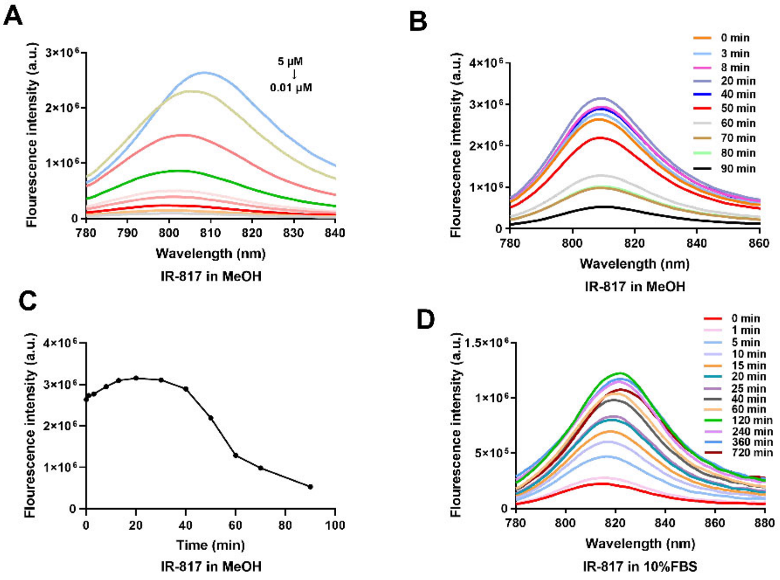

Figure S4. (A) The fluorescence intensity was plotted versus with different concentration (0.01-5 μM) of IR-817 in MeOH. (B) Fluorescence intensity of 5 μM IR-817 in MeOH over time. (C) Fluorescence instability of IR-817 in MeOH. (D) Fluorescence intensity of 5 μM IR-817 in 10% FBS over time.

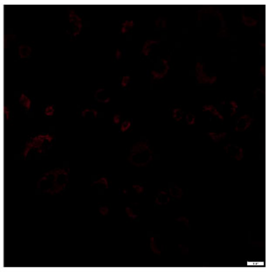

A549

Figure S5. Fluorescence image of 5 μM IR-817 co-incubated with A549 cells for 1 h. Scale.

bars, 20 μm.

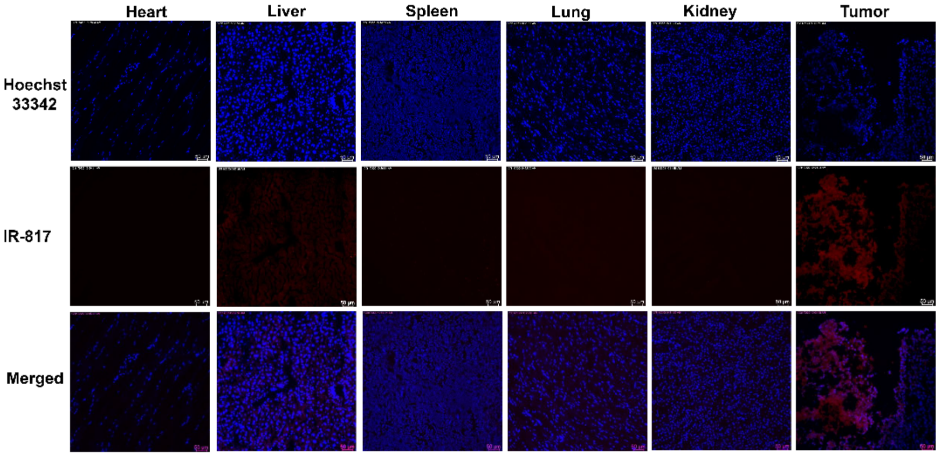

Figure S6. C57BL/6J mice with B16-F10 subcutaneous tumor xenografts were subjected to histopathologic analysis after a single-dose intravenous administration of IR-817 at 5 mg/kg for 12 h. Organs and xenografts were imaged by fluorescence microscope. Scale bars, 50 μm.

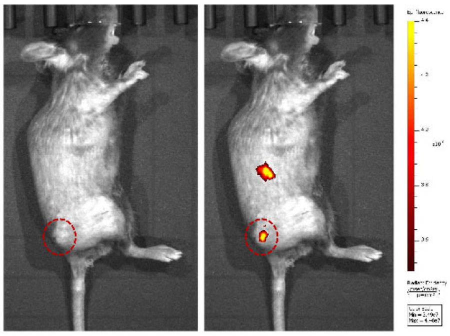

Figure S7. *In vivo* imaging of the subcutaneous tumor mice model after tail vein injection 48 h of IR-817 (5mg/kg).

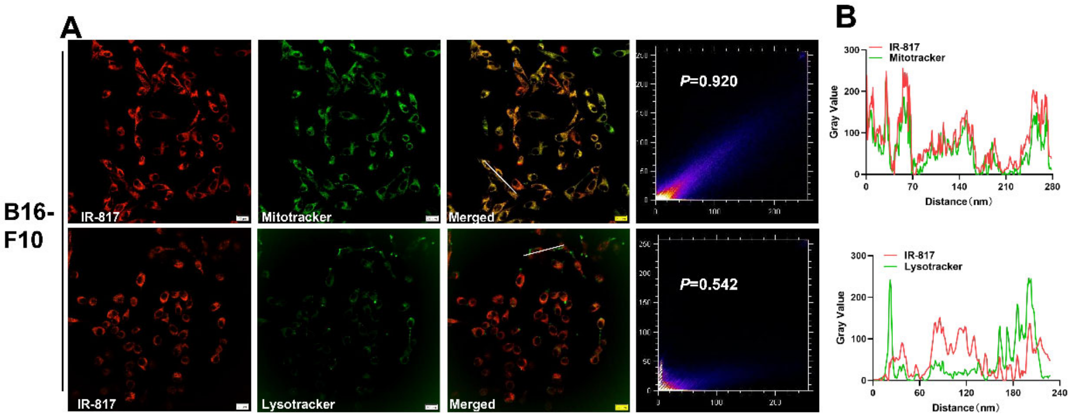

Figure S8. (A)Fluorescence co-localization of IR-817 and Mitotracker Green or Lysotracker Green in B16-F10 cells. (B) Normalized intensity profiles of co-localized image along the pixels marked by the solid line.

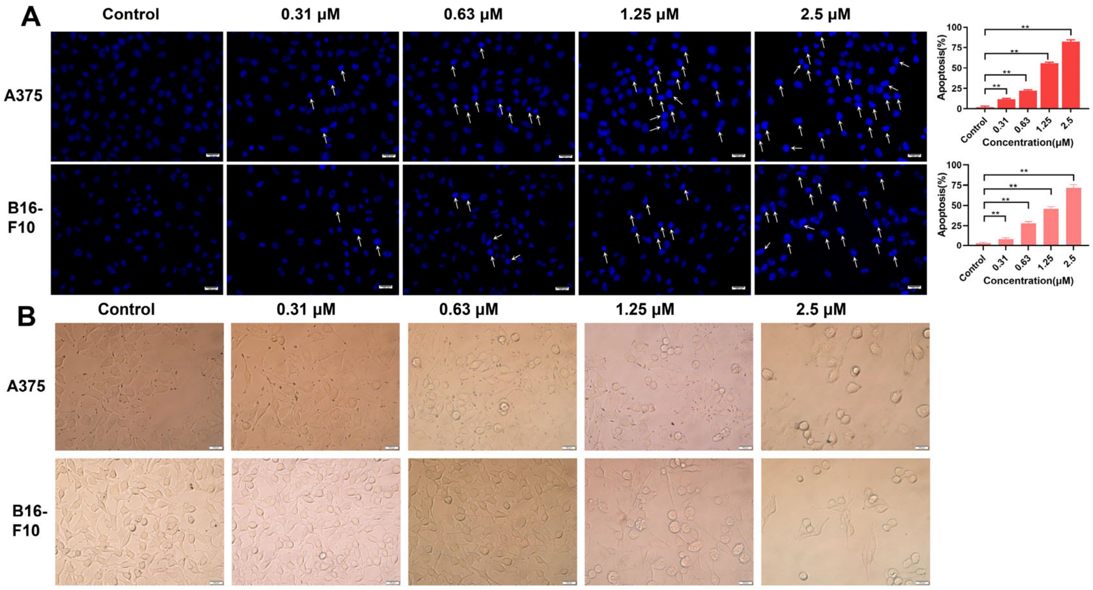

Figure S9. (A) The nuclear changes of A375, B16-F10 were observed after Hoechst 33342 staining (×400 magnification, scale bar =100 μm). The arrow refers to apoptotic cells. Each value was mean ± SD, n = 3. ** *p* < 0.01. (B) Photographs showing morphological changes in A375 and B16-F10 cells with different concentrations of IR-817 for 24 h.

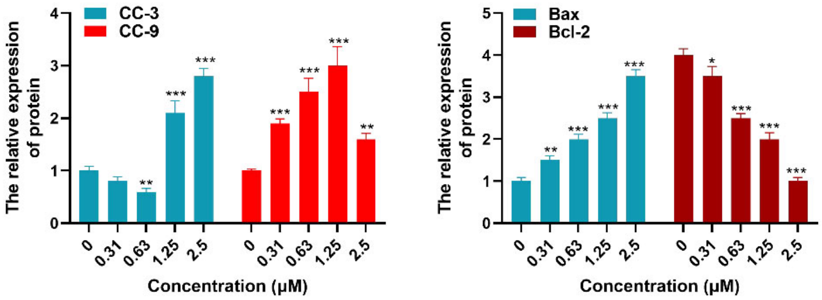

Figure S10. Relative protein expression levels of CC-3, CC-9 (35KDa), Bax and Bcl-2 in B16-F10 cells after different treatments analyzed from Western blotting were shown in each bar. Each value was mean ± SD, n = 3. *** $p < 0.001$,** $p < 0.01$, * $p < 0.05$.

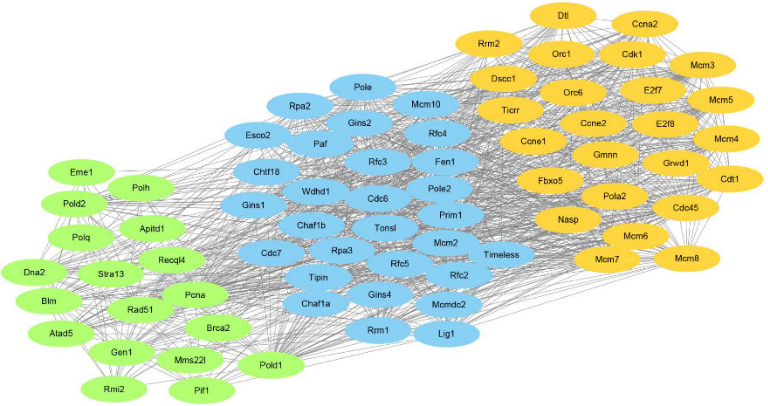

Figure S11. The PPI network complex of the 72 genes in the DNA repair pathway. The nodes meant proteins; the edges meant the interaction of proteins; three major clusters were identified by MCODE plug-in and labeled by green, blue and orange colors.

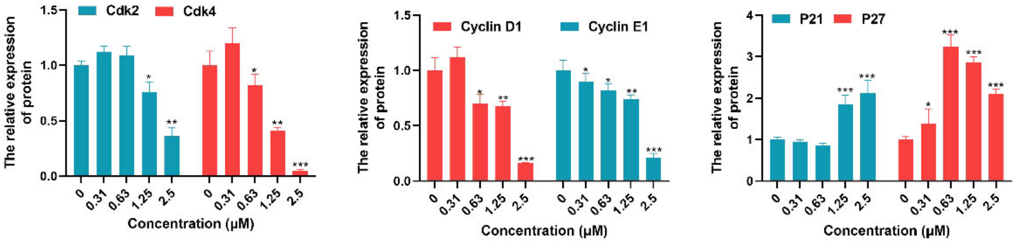

Figure S12. Relative protein expression levels of Cdk2, Cdk4, Cyclin D1, Cyclin E, P21 and P27 in B16-F10 cells after different treatments analyzed from Western blotting were shown in each bar. Each value was mean ± SD, n = 3. *** $p < 0.001$,** $p < 0.01$, * $p < 0.05$.
